# Estimating carbon sequestration in the piedmont ecoregion of the United States from 1971 to 2010

**DOI:** 10.1186/s13021-016-0052-y

**Published:** 2016-06-13

**Authors:** Jinxun Liu, Benjamin M. Sleeter, Zhiliang Zhu, Linda S. Heath, Zhengxi Tan, Tamara S. Wilson, Jason Sherba, Decheng Zhou

**Affiliations:** 1 Western Geographic Science Center, USGS, Menlo Park, CA 94025 USA; 2 USGS, 12201 Sunrise Valley Drive, Reston, VA 20192 USA; 3 USDA Forest Service, Northern Research Station, Durham, NH 03824 USA; 4 USDA Forest Service, Washington Office R&D, Washington DC, 20250 USA; 5 Arctic Slope Regional Corporation, Contractor to USGS EROS, Sioux Falls, SD 57198 USA; 6 San José State University Research Foundation, San José, CA 95112 USA; 7 Jiangsu Key Laboratory of Agricultural Meteorology, Nanjing University Of Information Science And Technology, Nanjing, 210044 China

**Keywords:** Land-use change, Carbon change, Piedmont ecoregion, IBIS model

## Abstract

**Background:**

Human activities have diverse and profound impacts on ecosystem carbon cycles. The Piedmont ecoregion in the eastern United States has undergone significant land use and land cover change in the past few decades. The purpose of this study was to use newly available land use and land cover change data to quantify carbon changes within the ecoregion. Land use and land cover change data (60-m spatial resolution) derived from sequential remotely sensed Landsat imagery were used to generate 960-m resolution land cover change maps for the Piedmont ecoregion. These maps were used in the Integrated Biosphere Simulator (IBIS) to simulate ecosystem carbon stock and flux changes from 1971 to 2010.

**Results:**

Results show that land use change, especially urbanization and forest harvest had significant impacts on carbon sources and sinks. From 1971 to 2010, forest ecosystems sequestered 0.25 Mg C ha^−1^ yr^−1^, while agricultural ecosystems sequestered 0.03 Mg C ha^−1^ yr^−1^. The total ecosystem C stock increased from 2271 Tg C in 1971 to 2402 Tg C in 2010, with an annual average increase of 3.3 Tg C yr^−1^.

**Conclusions:**

Terrestrial lands in the Piedmont ecoregion were estimated to be weak net carbon sink during the study period. The major factors contributing to the carbon sink were forest growth and afforestation; the major factors contributing to terrestrial emissions were human induced land cover change, especially urbanization and forest harvest. An additional amount of carbon continues to be stored in harvested wood products. If this pool were included the carbon sink would be stronger.

## Background

Increasing concentrations of carbon dioxide (CO_2_) in the atmosphere is a major cause of global warming. Major terrestrial CO_2_ emissions have been found where humans have disturbed the land by deforestation and agricultural practices [1, 2]. Because both forest and agricultural ecosystems are critical components of terrestrial C sequestration, many intensive observation and modeling studies have been undertaken to quantify ecosystem C change and C sequestration potential. Existing research shows forest ecosystems in the United States have been acting as C sinks, varying from 0.3 to 4 Mg C ha^−1^ yr^−1^ [3–11]. While forest harvest and natural disturbance lower forest C sequestration potential, forest rotation processes and natural recovery could make a forest system C neutral or a C sink if given enough time for recovery [12–14]. Studies of agricultural systems in the United States suggest that land-use changes caused severe soil organic carbon (SOC) loss from 1850 to 1960; but since the 1960s, improved farming practices (e.g., no-till) and increased C return to the soil have caused SOC to stabilize or possibly increase in some areas [15, 16]. Simulations of forest and agricultural ecosystems have produced large uncertainties regarding spatial and temporal variability of carbon dynamics, and identification of the driving forces of change [17–22]. Most uncertainties originate from difficulties in quantifying the impacts of disturbances and environmental variables. Land-use and land-cover change (LUCC) is a major disturbance factor, which strongly influences carbon budget calculations [2, 21, 23–25]. However, it has been a challenge to detect and quantify the dynamic nature of LUCC over large areas [21, 26, 27]. In the past, LUCC information in large-scale carbon sequestration modeling was not well developed, mainly due to the lack of consistent data describing changes in land use and land cover.

Several LUCC-oriented carbon studies have been conducted based on reconstructed LUCC histories [20, 23, 28–32]. However, these land cover change histories were usually averaged at a coarse spatial scale. Additionally, remote sensing is often used to detect tree cover loss at the time of disturbance whereas detection of regeneration following harvest is delayed. For agricultural ecosystems, previous research was usually at a local scale and under experimental control [33]. Quantifying the magnitude and spatial variation of regional carbon sources or sinks was found to be difficult because of the high spatial variability in site conditions and the diversity of human management.

More recently, high-resolution land-change datasets, such as the US Geological Survey’s Land Cover Trends (LCT) dataset have become available [34, 35]. The LCT data is the longest temporal record of consistent, empirically-derived, high resolution LUCC data available for the US at present. This ecoregion-based assessment of land-use change was guided by a nationally consistent study design including mapping, statistical methods, field studies, and analysis [26, 34, 36]. The sequential LUCC maps for the Piedmont ecoregion have a 60-m spatial resolution, a much finer resolution than any previously used in C accounting for the conterminous United States [19, 20, 37].

In this study, we report the use of the Integrated Biosphere Simulator (IBIS) in simulating carbon dynamics of forest and agricultural ecosystems in the Piedmont ecoregion from 1971 to 2010. The 60-m resolution 1973–2000 LCT data were used to generate 960-m annual land cover change maps from 1971 to 2010 (see more details in the “Methods” section). We focused on the effective use of the annual maps in analyzing land-change effects on biomass and soil C, as well as harvested C trends related to forest cover change.

## Methods

### The Piedmont ecoregion and LUCC detection

The Piedmont is a hilly, transitional ecoregion between the flatland near the Atlantic coast and the mountainous Appalachian ecoregions of the eastern United States. It has an area of 165,460 km^2^, as delineated by EPA level III ecoregions [38]. Annual precipitation ranges from 1100 to 1400 mm. Average annual minimum temperature ranges from 7 to 12 °C, and maximum temperature ranges from 20 to 25 °C.

**Fig. 1:**
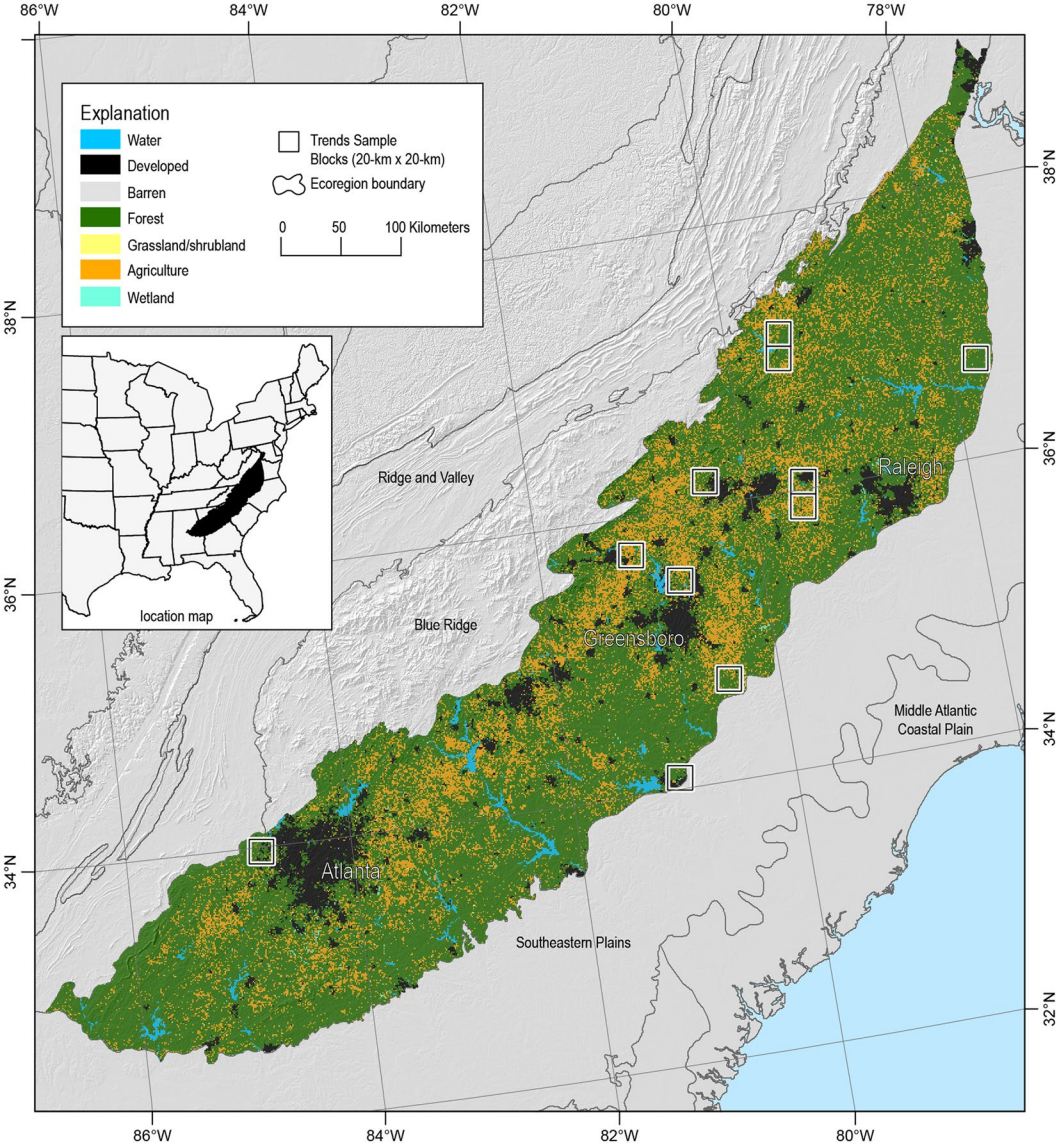
The Piedmont ecoregion from the 1992 National Land Cover Dataset (Vogelmann et al. [39]) modified to the ten land use and land cover classes of the USGS Land Cover Trends Project. *Square boxes* indicate the eleven 20 km^2^ land cover change sampling blocks at 60 m spatial resolution

The Piedmont was an important farming region in the 19th century, but during the 20th century, forestry and land development became more competitive uses of land. In recent decades, the Piedmont has had a relatively fast land conversion rate compared with other ecoregions in the eastern US. Nearly 15 % of land in the Piedmont converted to a different cover type between 1973 and 2000 [34]. The LCT land cover change quantification was based on interpretation of land change across eleven 20-km by 20-km sample blocks randomly selected using a stratified randomsampling design [26, 36]. Imagery from the Landsat archive was manually interpreted for each sample block at five dates (1973, 1979, 1986, 1992, 2000). The classified maps include ten land use and land cover categories: water, developed, mechanically disturbed (clear cutting), mining, naturally barren (vegetation cover less than 10 %), forest, grassland/shrubland, agricultural land, wetland, and non-mechanically disturbed (i.e., forest cover loss attributed to fire, flooding, or disease) (For more details, see [26, 34]. The sample block locations are shown in Fig. 1. An example of land transition rates between 1992 and 2000 is listed in Table 1. Overall, from 1973 to 2000, the percentage of forest area decreased from 61 to 56 %, agricultural area decreased from 25 to 24 %, and the combined developed and mining areas increased from 11 to 17 % of the ecoregion area.

**Table 1:** Piedmont ecoregion land cover transition rates between 1992 and 2000

	Water	Developed	M.disturbed	Mining	Nat.bare	Forest	Gra/shr	Agri.	Wetland	N.M.dist.	Total
Water	*3576.9*	4.0	21.4	0.0	0.0	210.2	0.0	93.2	31.3	0.0	3937.1
Developed	11.2	*24,013.1*	172.9	9.3	0.1	2262.8	1.2	653.1	4.3	0.0	27,128.1
M.disturbed	0.0	0.0	*92.8*	0.0	0.0	3176.9	0.5	16.6	16.5	0.0	3303.2
Mining	0.0	0.3	0.9	*315.0*	0.0	64.7	0.0	25.3	0.0	0.0	406.3
Nat.bare	0.0	0.0	0.0	0.0	*3.9*	0.0	0.0	0.0	0.0	0.0	3.9
Forest	23.9	2.8	3669.0	1.1	0.0	*87,101.2*	40.2	313.5	0.3	0.0	91,151.9
Grass/shrub	0.0	0.0	30.1	0.0	0.0	8.6	*19.1*	10.1	0.0	0.0	68.0
Agriculture	0.7	0.0	154.3	0.0	0.3	557.7	0.0	*37,509.3*	0.1	0.0	38,222.3
Wetland	0.0	0.0	14.8	0.0	0.0	0.0	0.0	0.0	*1224.2*	0.0	1239.0
N.M.dist.	0.0	0.0	0.0	0.0	0.0	0.0	0.0	0.0	0.0	*0.0*	0.0
Total	3612.7	24,020.3	4156.2	325.4	4.3	93,382.1	61.1	38,621.2	1276.6	0.0	165,459.9

Areas summed by row indicate total land area in 2000; Areas summed by column indicate total land area in 1992. Area units: km^2^. Total forest area in 1992 was 93,382.1 km^2^. Forest to developed land (urban) conversion between 1992 and 2000 was 2262.8 km^2^. Forest remaining as forest was 87,101.2 km^2^. Forest to agriculture conversion was 557.7 km^2^. Total forest area in 2000 was 91,151.9 km^2^, of which 313.5 km^2^ was converted from agriculture

Values italicized within the table represent land use and land cover remaining constant

### IBIS model framework and calibration

The integrated biosphere simulator (IBIS) [40, 41] is a physically consistent modeling framework that follows basic rules of physics, plant physiology, and biogeochemistry. The original model combined features of a mechanistic model of canopy photosynthesis [42], a semi-mechanistic model of stomatal conductance [43], an algorithm on phenology [44], and several soil biogeochemical models [45–47] in a single application. IBIS has the ability to simulate major land surface processes, canopy physiology, vegetation phenology, long-term vegetation dynamics, ecosystem productivity, and carbon cycling.

**Fig. 2:**
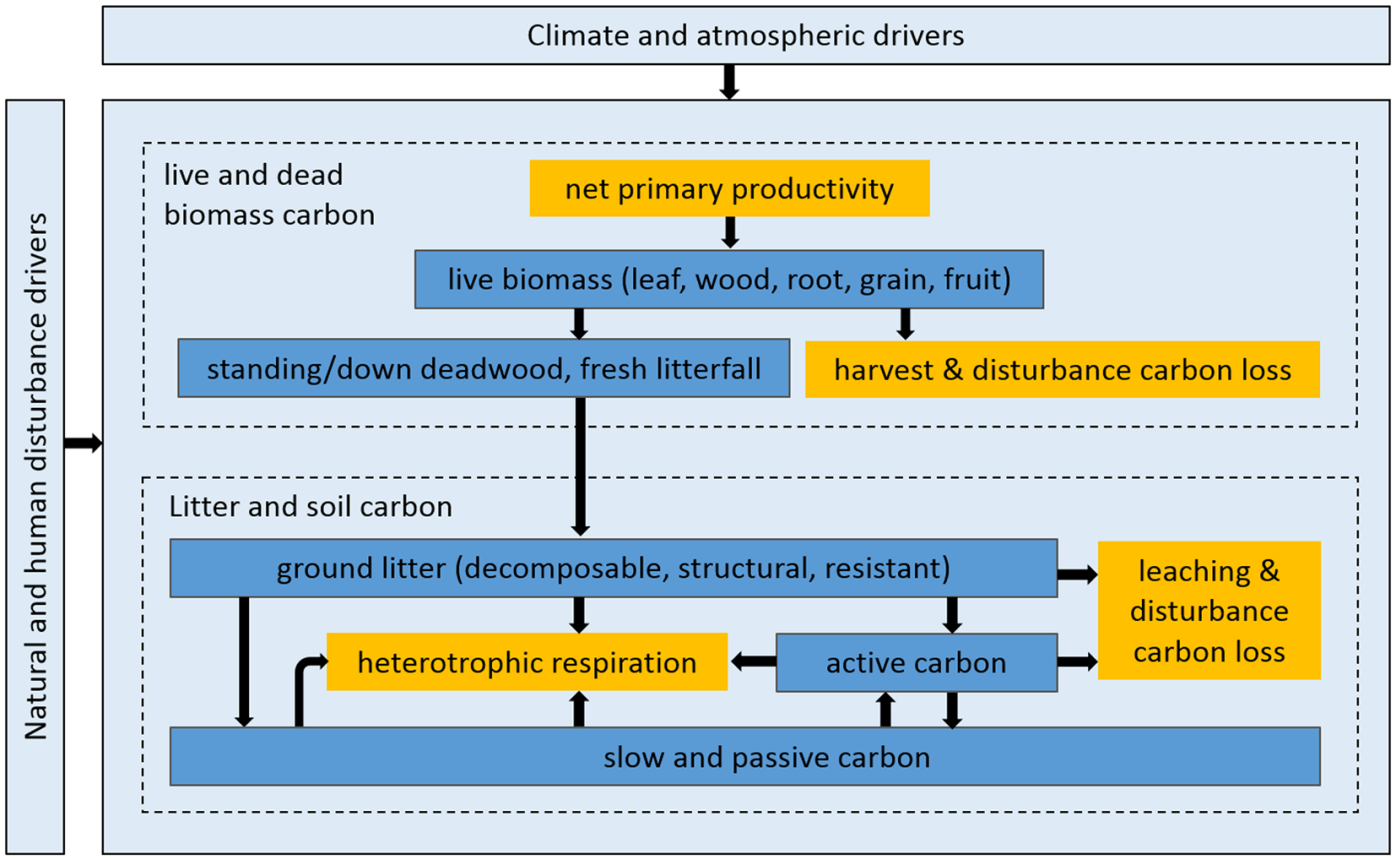
Overall ecosystem carbon cycling process simulated in IBIS. *Blue boxes* indicate carbon pools; *orange boxes* indicate carbon inputs and outputs to the ecosystem; *arrows* show carbon flow between pools. Climate and disturbance control the ecosystem productivity, respiration, and carbon removals

A modified version of IBIS included nitrogen (N) controls on the carbon cycle [48], land-use and land-cover change, and wildland fire effects [49], and Methane (CH_4_) emission [50]. Figure 2 shows the major C pools, fluxes and flow pathways simulated by the IBIS model. The input C flux of the ecosystem is the net primary productivity (NPP), which is calculated from climate, soil and vegetation conditions. The output C flux includes woody biomass harvest, crop harvest, fire combustion, heterotrophic respiration and C leaching. Climate drivers mainly determine the ecosystem NPP and respiration calculations while disturbance (LUCC) events mainly affect the C removal and reset the vegetation C pools.

**Fig. 3:**
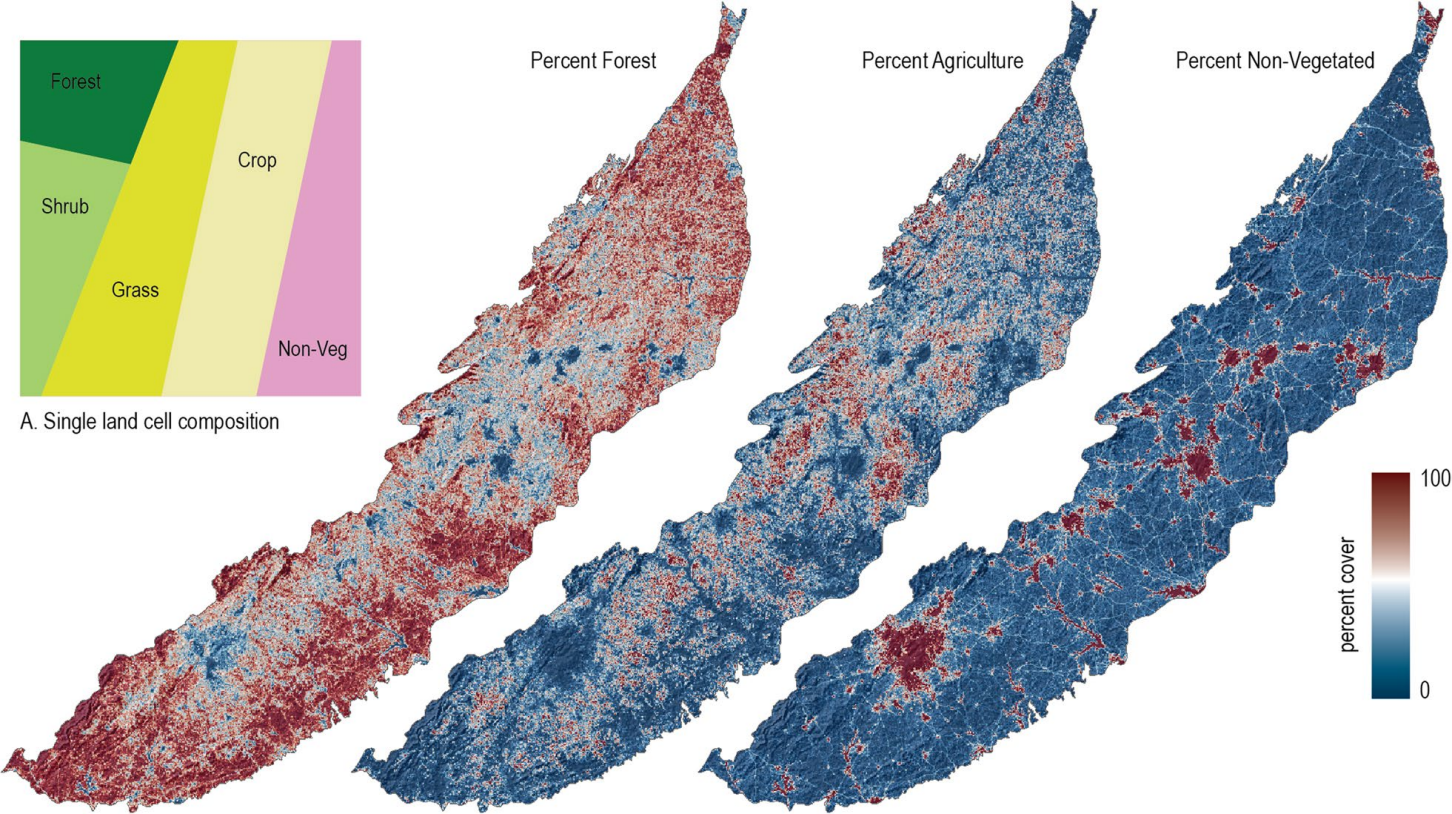
Conceptual IBIS single land pixel composition and the distribution and percentages of forest land, agricultural land and non-vegetation cover in the Piedmont ecoregion based on data obtained from the LandFire Project aggregated to 960-m from 30-m

Process-based biogeochemical models like IBIS usually simulate over an hourly time step. Therefore decade- and century-long simulations for a large region would take a significant length of time (weeks to months) if running at a very high spatial resolution (e.g. 60-m). Therefore, IBIS has been recently enhanced in several aspects, including: (1) program recoded to support parallel computing using Message Passing Interface (MPI) on super-computers; (2) treatment of fractional vegetation cover within a single land pixel in order to use the newly available higher resolution LUCC products; (3) spatial scalars for tree biomass growth and crop grain production at county level to deal with diverse geography. The IBIS conceptual land pixel includes multiple plant functional types, each competing for light, moisture and nutrient during simulation (Fig. 3A). The existing forest and agricultural cover fraction maps for the Piedmont ecoregion were built from the 30-m vegetation canopy maps from the LandFire Project and aggregated to 960-m (Fig. 3). Most of the 960-m pixels are a mixture of several land cover types. The modified IBIS tracks the percent area of each land cover type within each land pixel. When a LUCC or disturbance event (e.g., reforestation, deforestation, urbanization, etc.) occurs, cover fractions are transferred between relevant land cover types.

The current IBIS version deals with 11 types of disturbances: (1) fire, (2) logging, (3) deforestation to grass/shrub, (4) deforestation to cropland, (5) afforestation from grass/shrub, (6) afforestation from agriculture, (7) urbanization from forest, (8) urbanization from grass/shrub, (9) urbanization from cropland, (10) agricultural expansion (grass/shrub to cropland), (11) agricultural contraction (cropland to grass/shrub). Logging and fire events may only trigger C removal and additional tree mortality; forest cover fraction will remain unchanged, allowing for forest regrowth. Other types of disturbance will remove carbon from the landscape and also alter land-cover fractions. For example, forest to cropland transition (deforestation) will re-allocate previous forest cover fraction to the cropland cover fraction, and remove all forest carbon from the landscape. As a result, the following simulation year will have no forest productivity, but more crop productivity due to crop area fraction increase.

In addition to disturbances detectable through remote sensing methods, we also consider the less easily detectable events like forest thinning activities. Forest thinning rate is calculated using recent annualized forest inventory data collected by the US Forest Service, Forest Inventory and Analysis Program [51, 52]. Thinning activity is loosely defined as the cutting-related biomass carbon loss of less than 50 % during two consecutive observation periods (around 5 years) in order to make the overall thinned area percentage (i.e., 61 % of the total forest cutting area) in agreement with earlier estimates [53, 54]. For the Piedmont ecoregion, the annual thinning rates in terms of total live aboveground biomass carbon range from 0.31 to 1.16 % (average 0.81 %) in different counties. Forest thinning removes an amount of tree carbon which is not usually a detectable change in forest cover fraction.

For this study, the following LUCC were considered: logging, deforestation (forest to agriculture conversion), afforestation (agriculture to forest conversion), agriculture contraction (agriculture to grassland conversion), agriculture expansion (grassland to agriculture conversion), and urbanization (forest to urban, grassland to urban, and agriculture to urban).

Carbon output variables of the IBIS model include live and dead biomass, soil organic carbon, carbon losses from disturbance, as well as net primary productivity (NPP) and net biome productivity (NBP). In this study, we used the dominant vegetation cover (i.e., forest, agriculture, shrub, and grass) to summarize carbon variables because most land pixels are mixed with more than one cover type. Statistics for forest land pixels usually include a certain amount of other vegetation types. Similarly, agricultural land summaries may also contain a small amount of forest and other vegetation covers.

IBIS uses biome level plant functional types (PFT) to represent major vegetation groups, which are coarsely defined in the model based on climate conditions. Some stand or landscape level carbon control factors for forest systems are not considered in the current version of IBIS, such as tree species, age class and stem density. Similarly, the modeled crop system only includes two generic crop PFTs (C3 and C4 crops). This makes site level model calibration difficult.

To validate the model, a county level calibration procedure was developed. For forest, 1-km MODIS forest NPP for 2001–2005 was averaged at the county level and compared with IBIS simulated forest NPP. Then, an adjustment scalar was introduced. The scalar was assumed to help in dealing with unknown environmental factors (e.g., species, age, stem density). For crops, without considering details like crop species, irrigation, fertilization, and double cropping in IBIS, we used the county level USDA NASS crop yield statistics and IBIS grain yield to generate a county level grain yield scalar, which partly reflects the human activity difference by geolocation. The scalars were used to modify the Maximum Rubisco-limited rate of carboxylation (Vmax) of related PFTs (forest or crop) in a new IBIS run. In addition to NPP and grain yield calibration, simulated forest live biomass at 100 years of age was also calibrated. Forest growth curves from the Carbon On Line Estimator (COLE) [55, 56] were used as the general forest growth references to be compared with IBIS growth curves.

### Data sources

Land-change information from the LCT project, wildland fire scar and burn severity data from the Monitoring Trends in Burn Severity project [57], and vegetation canopy percentage and vegetation height information from the Landfire project [58] were the key variables for calculating vegetation fraction and biomass on each land pixel, as well as the effects of logging, deforestation, afforestation, urbanization, agricultural expansion and contraction, and wildland fire on C changes. These 30- and 60-m datasets were aggregated to 960-m resolution for this study.

We extended LUCC mapping to include 1971–1972 and 2001–2010, using the LCT land conversion rates of 1973–1979 and 1992–2000, respectively. We used the LUCAS model [59] to create an annual time-series of land use and land cover maps for the period 1971–2010. The 2001 National Land Cover Database (NLCD) [60] was used as the starting point, and changes were backcasted into the historical period based on (1) rates of change between the four temporal periods from the LCT data, and (2) adjacency rules which prescribed change to occur adjacent to existing cells (also see Daniel et al. [61]). For the period 2001–2010, the LUCAS model was run forward in time using the LCT rates from the 1992–2001 period. All simulations were done on an annual timestep, at a spatial resolution of 1-km.

Global atmospheric CO_2_ concentration trends were based on observed data [62]. Spatially heterogeneous atmospheric CO_2_ measurements from the Scanning Imaging Absorption Spectrometer for Atmospheric Cartography (SCIMACHY) from ENVISAT (0.5° resolution, 2003–2009) were converted to monthly surface CO_2_ concentration to produce an average monthly CO_2_ difference to global average CO_2_ map [63], which was used to adjust the CO_2_ fertilization calculation.

The PRISM 4-km resolution monthly precipitation and temperature data from 1971 to 2010 were used as the main climate driver. Other climate variables, such as relative humidity and wind speed, were included as monthly normals across the 1961–1990 time series. The SSURGO soil carbon and texture (960 m resolution, ~2000) dataset was used for initial soil conditions [64]. The soil profiles contain up to six depth layers (to 7, 15, 25, 50, 100, 200 cm depths) and include sand, silt, and clay fractions for each.

For the forest growth calibration, summary results for each FIA survey unit were downloaded from the COLE website (http://www.ncasi2.org/COLE/). The 100 year average forest biomass growth values were used to calibrate IBIS simulated 100 year total tree biomass growth. For agricultural ecosystems, county-level grain yield statistics were downloaded from the USDA National Agricultural Statistics Service (NASS) website (https://www.quickstats.nass.usda.gov/) and used to calibrate simulated cropland grain yield.

## Results

### Land cover change trends and annual maps

**Fig. 4:**
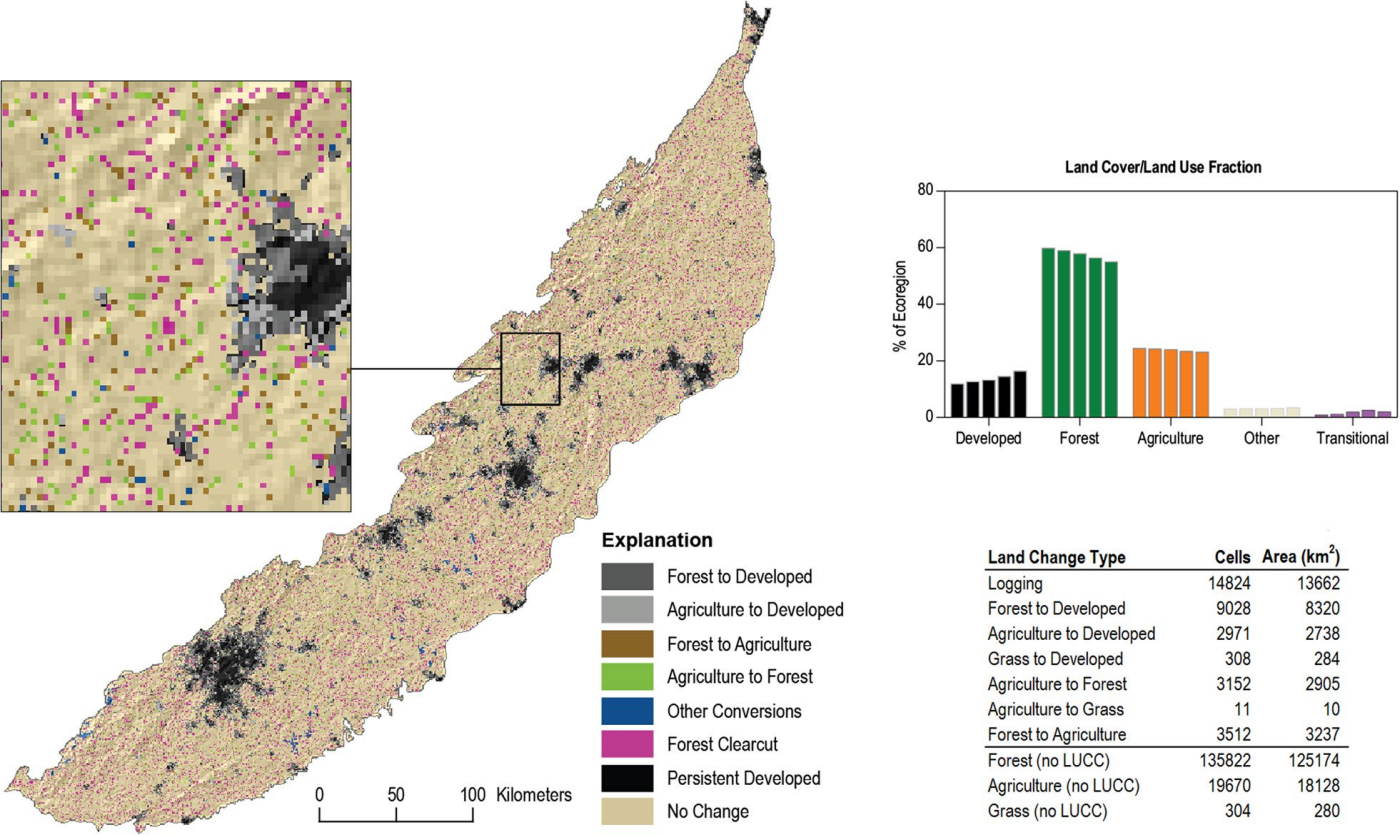
Total land cover change between 1971 and 2010 in the Piedmont ecoregion. Map is at 960-m resolution. Changing pixels indicate the simulated locations of land cover change

Statistics showed net decreases in forest (−4.76 %) and agricultural (−1.27 %) land area and net increases in urban (4.49 %) and disturbance areas (1.14 %) between 1973 and 2000. Grassland, wetland, and water bodies had much lower change rates (<0.3 %) during this period. The calculated annual land change amounts for the IBIS C simulation (1971–2010) were mapped at 960 m spatial resolution. Rotational logging accounted for the most area over the study period. The percentage of forest area logged versus total forested area increased from 1.43 to 3.62 % over the simulation period, indicating an intensification in the rotational logging cycle, in addition to increasing rates of deforestation due primarily to urbanization. The overall land change statistics and mapping are shown in Fig. 4.

### Regional ecosystem carbon trends

**Fig. 5:**
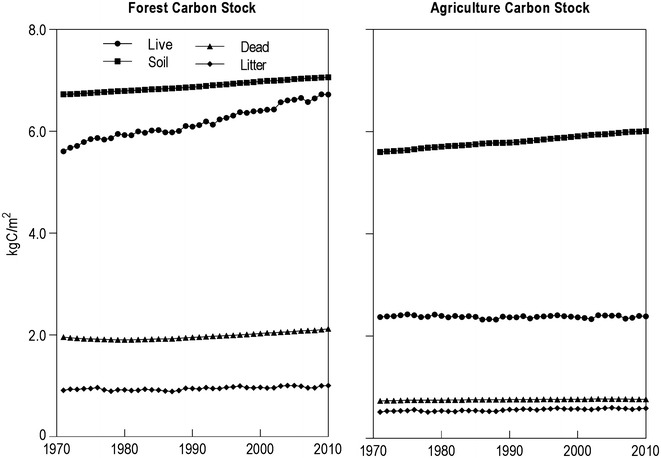
Trends of major carbon pools in forest and agricultural ecosystems in the Piedmont ecoregion between 1971 and 2010. The major pools are living biomass (“live”), soil organic carbon (“soc”), dead biomass (“dead”), and litter

Changes in the major C pools of forest and agricultural ecosystems are shown in Fig. 5. For forest ecosystems (forest dominant land pixels), live biomass C increased from 5.61 to 6.72 kg C m^−2^ over the 40-year simulation period. SOC increased from 6.73 to 7.06 kg C m^−2^. Dead wood and litter carbon pools increased slightly from 2.87 to 3.13 kg C m^−2^. Overall, the forest ecosystem was a net C sink of 0.025 kg C m^−2^ yr^−1^. The agricultural ecosystem was a small C sink during the simulation period. Live biomass C remained stable around 2.38 kg C m^−2^; SOC increased from 5.60 to 6.02 kg C m^−2^, and litter and dead biomass increased from 1.25 to 1.36 kg C m^−2^ over the 40-years. The agricultural lands are not made up of pure land cover pixels and still include some forest cover. The average annual C sink was only 0.003 kg C m^−2^ yr^−1^.

**Fig. 6:**
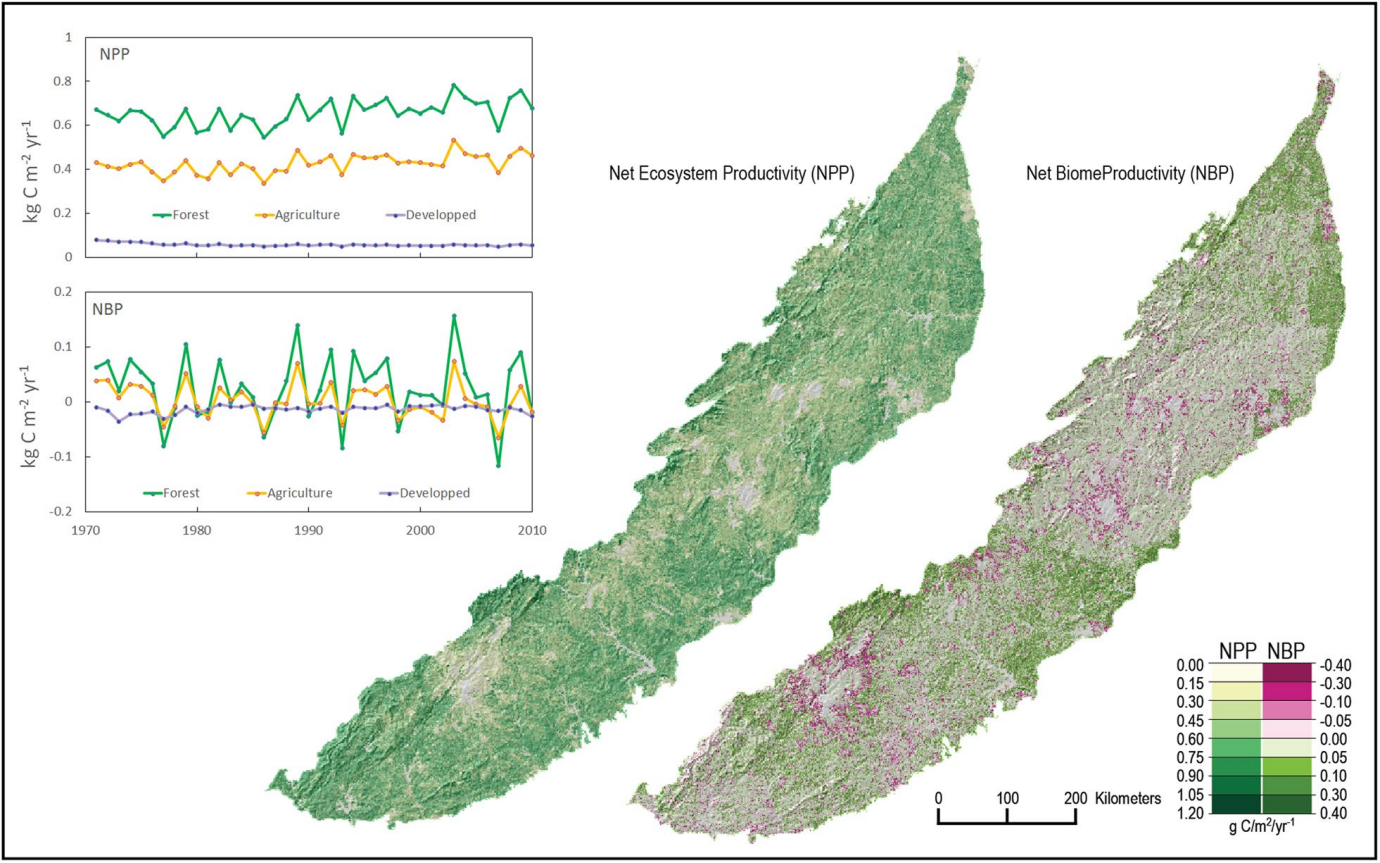
Trends and spatial distribution of net primary productivity (NPP) and net biome productivity (NBP) in the Piedmont ecoregion between 1971 and 2010. The maps show the average over 40 years, expressed in kg C m^−2^ yr^−1^

In terms of ecosystem productivity, both forest and agricultural ecosystems showed some inter-annual fluctuations of NPP and NBP over the study period (Fig. 6). Average forest NPP increased from 0.61 to 0.71 kg C m^−2^ yr^−1^. Agricultural ecosystem NPP increased from 0.39 to 0.46 kg C m^−2^ yr^−1^.

Logging removal (rotational clear-cutting) from forest ecosystems averaged 0.012 kg C m^−2^ yr^−1^, with the 1970s exhibiting the lowest harvest rates (0.006 kg C m^−2^ yr^−1^), and the late 1980s and early 1990s exhibiting the highest (0.020 kg C m^−2^ yr^−1^). The estimated forest thinning removal averaged 0.048 kg C m^−2^ yr^−1^ during the study period. Carbon removal following forest to agriculture conversion and forest to urban conversion was about 0.011 kg C m^−2^ yr^−1^. Due to the non-pure land pixels at the 960 m spatial resolution, there was also agricultural removal (grain and straw) in forest areas, which increased from 0.017 to 0.021 kg C m^−2^ yr^−1^. The forest NBP averaged 0.025 kg C m^−2^ yr^−1^. Carbon removal from the agricultural system (grain and straw) increased from 0.075 to 0.118 kg C m^−2^ yr^−1^ over the study period. An estimated 0.001 kg C m^−2^ yr^−1^ was lost from logging removals in agricultural ecosystems due to non-pure land pixels. The agricultural ecosystem NBP averaged about 0.003 kg C m^−2^ yr^−1^.

For the whole ecoregion, the 40 year average NBP was 3.32 Tg C on a valid calculation area of 157,415 km^2^, with forest dominant ecosystems accounting for approximately 3.32 Tg C (~133,380 km^2^, averaged between 1971 and 2010) and agricultural dominant ecosystems accounting for approximately 0.06 Tg C (~19,522 km^2^). Newly established urban land lost carbon at a rate of 0.014 kg C m^−2^ yr^−1^, and totalled 0.05 Tg C per year (~4497 km^2^, averaged between 1971 and 2010). The spatial distribution of the 40 year average NPP and NBP are also displayed in Fig. 6.

### Land cover change impact

**Fig. 7:**
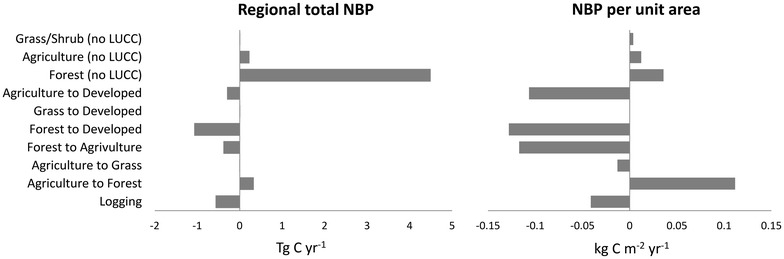
Land cover change impact on net biome productivity (NBP) in Piedmont ecosystems

We analysed the simulated NBP map series to show the consequences of LUCC on the C cycle (Fig. 7). Piedmont forest ecosystems had an average NBP of 25 g C m^−2^ yr^−1^ over the 40 year period. Forest dominant ecosystems without land conversions had an average NBP of 36 g C m^−2^ yr^−1^, while forests that had undergone rotational clear-cutting, deforestation to agriculture land, or clearing for urbanization showed C losses at 41, 117 and 128 g C m^−2^ yr^−1^, respectively. On the other hand, afforestation locations gained 112 g C m^−2^ yr^−1^ on average. Cropland had an average NBP of 3 g C m^−2^ yr^−1^ over the 40 year period. Without land conversions, cropland had an average NBP of 12 g C m^−2^ yr^−1^. Agricultural land converting to grassland led to C loss at a rate of 12 g C m^−2^ yr^−1^. Additionally, conversions of agricultural land to urban also led to C loss, 107 g C m^−2^ yr^−1^, mainly due to removal of trees on non-pure agricultural lands. The regional total NBP for each LUCC type were also displayed on Fig. 7.

### Carbon budget of Piedmont ecoregion

**Fig. 8:**
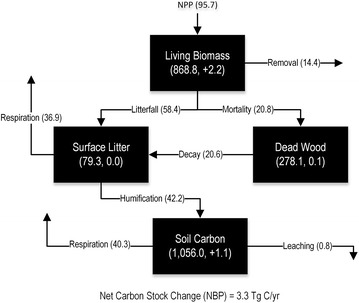
Overall average annual Piedmont ecoregion carbon budget from 1971 to 2010 expressed in teragrams (Tg) of C per year. *Blocks* are carbon pools, with average pool size and average annual change amount. All other *names* and *numbers* are carbon fluxes

The NBP of forest and agricultural lands in the Piedmont ecoregion varied annually as indicated in Fig. 6. The Piedmont ecoregion has 170,806 simulated 960 m resolution land pixels and with 1 kg C m^−2^, that equals 157.4 Tg C (1 Tg = 10^12^ g = 1 million tons) for the whole region. Figure 8 shows the average annual C budget of the Piedmont over the 40 year period. The four aggregated carbon pools show that Piedmont soil had the largest carbon storage and also had the largest carbon increase. Live biomass carbon increase was small due to removal and mortality. The major carbon emissions were litter and soil respiration. Overall, NBP for the Piedmont Ecoregion was a small net sink at a rate of 3.3 Tg C per year.

### Model validation and uncertainty

**Fig. 9:**
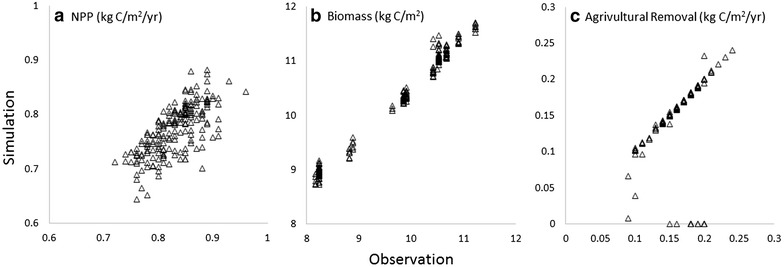
IBIS model validation against remote sensing and field survey data at the county level. **a** Simulated forest NPP against MODIS NPP product; **b** Simulated 100 year forest tree biomass against FIA based COLE database; **c** Simulated agricultural harvest carbon removal (grain + hay + straw) against USDA NASS crop harvest data. Each data point represents a county in the Piedmont ecoregion (total 198 counties)

This study used county level observations and model results from other sources to adjust model outputs. We used FIA COLE data and USDA NASS grain yield data as field truth datasets. The MODIS NPP product (based on remote sensing and modelling) was used once to generally adjust the NPP range because the IBIS model does not consider tree species, local management, etc., which were potentially well captured by remote sensing. We avoided overfitting because IBIS has its own NPP algorithm. Figure 9 shows the adjustment.

In general, IBIS NPP matched with MODIS NPP. Since this was not a pixel level comparison, IBIS forest NPP still maintained its spatial variability driven by climate, soil, vegetation and disturbance. Forest biomass and crop yield output fit closely to field observation data because multiple iterations were run on the related spatial scalars. Again, the calibration was done at the county level, therefore the output maintained spatial heterogeneity.

## Discussion

### Total ecosystem C budgets

Our current estimate of NPP for the Piedmont forests was 656 g C m^−2^ yr^−1^ (6.56 Mg C ha^−1^ yr^−1^). This result is higher than a MODIS NPP estimate of 550 g C m^−2^ yr^−1^ (5.5 Mg C ha^−1^ yr^−1^) for temperate forests [65]. However, our forest NPP estimation is close to MODIS NPP estimation in the Piedmont region as indicated in our calibration result (Fig. 9). In our simulations, the average annual forest biomass C growth (before harvesting) was about 1.2 Mg C ha^−1^ yr^−1^. Assuming 70 % of this growth is above-ground biomass C growth (0.84 Mg C ha^−1^ yr^−1^), then it is very close to Caspersen’s [66] estimate of average US forest above-ground biomass C increase (0.8 Mg C ha^−1^ yr^−1^). However, after adding the carbon removal amount, our Piedmont forest NBP from 1971 to 2000 was only 0.25 Mg C ha^−1^ yr^−1^. This estimate is significantly lower than that reported by Hurtt et al. [20], who estimated a forest C sink, in the 1980s, for all the United States, of about 0.9 Mg C ha^−1^ yr^−1^. It is also lower than the report of Tan et al. [67] focusing on US federal lands, which estimated a rate of about 0.6 Mg C ha^−1^ yr^−1^. It is much lower than a study of the nearby Appalachian forests, where NBP was estimated at approximately 1.8 Mg C ha^−1^ yr^−1^ [24], mainly due to more intensive forest harvesting in the Piedmont forests compared to the Appalachian forests. As previously indicated, forest thinning was considered in our simulations, which accounted for two times the amount of C removal compared to forest clear-cutting (including deforestation). Logging, thinning and deforestation were the major causes of Piedmont’s low C sink strength.

Agricultural ecosystem NPP averaged 425 g C m^−2^ yr^−1^ **(**4.25 Mg C ha^−1^ yr^−1^), and NBP was 3 g C m^−2^ yr^−1^ (0.03 Mg C ha^−1^ yr^−1^). Our NBP estimate was lower than controlled site level studies (e.g., [68], which reported a sink of 36 g C m^−2^ yr^−1^ for a complex crop rotation receiving both manure and chemical fertilizers). However, it is already understood that regional simulation results are typically lower than site-level results [69]. Especially when fractional cropland areas are considered, lower NPP and NBP is not unexpected. In addition, we applied a straw removal rate of 50 % in the study. This ratio was another factor influencing the C sink level on agricultural lands.

### Impact of LUCC

Although deforestation commonly led to a C source and reforestation led to a C sink as indicated in Fig. 7, the influence on the regional C budget depends on the total area affected. The total forest area of the US has been very stable at 2.98 million km^2^ during the past several decades, with a 0.1 % average fluctuation [70, 71]. During the 1990s and 2000s, US total forest land had increased by approximately 1 % of total US land area [72]. However, net forest area decrease (accounting for both deforestation and afforestation), in the Piedmont ecoregion, from 1971 to 2000, was about 5 % of the total land area and about 10 % of the total forested area. This would have a direct influence on the overall C budget. In this study, we applied a simple rule for vegetation regrowth following urbanization related land cover changes, i.e. allowing up to 15 % vegetation cover on newly generated urban lands. Urban lands on average were a C source of about 14 g C m^−2^ yr^−1^ (0.14 Mg C ha^−1^ yr^−1^).

The intensive stand-replacing disturbances occurring in the Piedmont ecoregion were a key factor leading to below-average NBP levels compared to other regions. Disturbed sites may need at least 20 years to regain the lost C [4, 14, 73]. In this study, the logging land pixels had an average NBP of −41 g C m^−2^ yr^−1^ over a 40 year period (average logging was about 20 years), indicating the recovery process may take even longer. On the other hand, forest thinning led to the largest amount of C removal in our simulation and represents an important source of uncertainty in modeling regional carbon dynamics.

For agricultural ecosystems, LUCC effects were linked to land conversions only. At present, we didn’t model other land management activities on cropland, such as fertilization and irrigation.

Combined, an estimated 14.4 Tg C was removed from ecosystems every year, of which 69 % (~9.9 Tg C) were from forest thinning and clear-cut. Some of the removed carbon continues to be stored in harvested wood products (HWP). However, dynamics of this important carbon pool were not considered in this study. If carbon storage in HWP were included the carbon sink would be stronger.

### LUCC data across scales

Currently, research by the USGS LandCarbon project [74] is underway to produce 30-m, annual, wall-to-wall land cover change maps at the national scale based on the Landsat 40 year data archive. The up-coming highly relevant LUCC map product will allow carbon models to make much better C estimations and better differentiate drivers from combined climate and land change interactions. However, using these high resolution land cover change data can be difficult for large regional C assessments. Aggregating the high resolution data to a coarser resolution would help complicated process-based models to use the LUCC information effectively. For even larger scale C modelling work, such as global C simulations, the LUCC information could be more inaccurate at coarse spatial resolution (such as 1–2°). New LUCC products such as the Global Change Assessment Model (GCAM) already focus on building a mixed land cover product. Therefore, using mixed land cover data in C models is a new and necessary approach for carbon accounting, especially for large region C assessments.

### Statistics of mixed land pixels

Although it is meaningful to use fractional land cover for carbon simulation in large regions, there exists the challenge of correctly interpreting LUCC and its effects. The use of forest dominant or agriculture dominant lands can potentially lead to confusion. For example, the Piedmont ecoregion has about 122,590 km^2^ of forest dominant land and 18,472 km^2^ of cropland dominant land. Yet this does not mean forest cover is 6.6 times larger than agricultural land cover. The Land Cover Trends data (pure land pixels) actually shows that forest area is about 2.4 times agricultural land area in the Piedmont. Therefore, alternative summary methods should be explored.

## Conclusion

The Piedmont ecoregion in the eastern US was estimated as a weak C sink with an average C gain of 3.3 Tg C yr^−1^. The overall per unit area C sink was 0.025 kg C m^−2^ yr^−1^, which is much smaller than the rates in the Appalachians and other eastern ecoregions. The major cause was the rapid human induced land-cover and land-use changes, especially forest logging, thinning, and urbanization. The method used in this study helps to quantify the overall human land use effect on the carbon budget and would be suitable at national and global scales when more detailed and consistent LUCC data becomes available.
